# Bilateral Pseudarthrosis of the Femoral Neck in a 25-Year-Old Male with Hereditary Hypophosphatemic Rickets

**DOI:** 10.1155/2014/312712

**Published:** 2014-05-15

**Authors:** Joris Anthonissen, Christian Ossendorf, Thomas Vetter, Björn Habermann, Pol M. Rommens

**Affiliations:** Department of Orthopaedics and Traumatology, University Medical Center, Johannes Gutenberg University of Mainz, Langenbeckstraße 1, 55131 Mainz, Germany

## Abstract

Hereditary hypophosphatemic rickets (HHR) is a rare disorder of renal phosphate wasting and the most common form of heritable rickets. Here, we report a case of an active 25-year-old male with HHR showing atraumatic bilateral femoral neck pseudarthrosis after 4 years of consecutive knee pain. A conservative therapy was administered, taking into account both the risks of surgical treatment and the little impairment even in the sport activities which the patient experienced.

## 1. Introduction

Hereditary hypophosphatemic rickets (HHR), also called X-linked dominant hypophosphatemic rickets or X-linked vitamin D-resistant rickets, is a rare X-linked dominant form of rickets that differs from most cases of rickets in that ingestion of vitamin D is ineffective [1]. It is associated with a mutation in the PHEX gene sequence (Xp.22) and subsequent inactivity of the PHEX protein [2]. The prevalence of the disease is 1 : 20,000 [3]. Clinical manifestations vary in severity, but patients most commonly present in childhood with bowing deformities of the legs. Progressive bowing and anteromedial rotational torsion of tibiae and short stature represent the predominant skeletal outcomes in growing children. With medical therapy, these abnormalities can be improved but usually do not entirely resolve [4, 5]. Metaphyseal changes of rickets are usually evident on radiographs. Osteomalacia (accumulation of unmineralized osteoid) is a characteristic of untreated HHR [6].

## 2. Case Report

A 25-year-old man presented with pain in both knees for four consecutive years and, since 3 months, bilateral groin pain and pain in the gluteal region, radiating to both knees.

The patient has been diagnosed with HHR as a child and has been on potassium phosphate and calcitriol tablets ever since. A typical bowing and anteromedial rotation of the tibia was corrected with tibial shaft osteotomies and Ilizarov fixators of both tibias about 10 years ago [7]. After these procedures, very slow healing of the bone with multiple revisions due to delayed union was observed.

**Figure 1:**
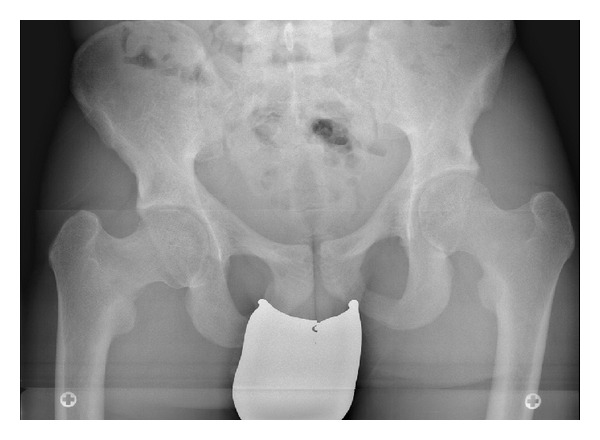
Standard pelvic radiograph taken 4 years ago showing no radiographic evidence of a fracture.

The patient has had pain in both knees for four years, more in the right than in the left knee. Initially, at 6 years of follow-up, approximately 4 years ago, MRI of both knees and pelvic X-ray did not reveal any pathological changes other than a strong coxa vara malformation of both hips (Figure 1). Pain was described by the patient as start-up pain that disappeared or bettered after walking with full weight bearing for a while. Since about 3 months, the pain began to concentrate on both hips radiating in both knees; the same start-up character of the pain was described. Upon questioning, the patient admitted that he has not been taking his medication regularly the last 6 months.

**Figure 2:**
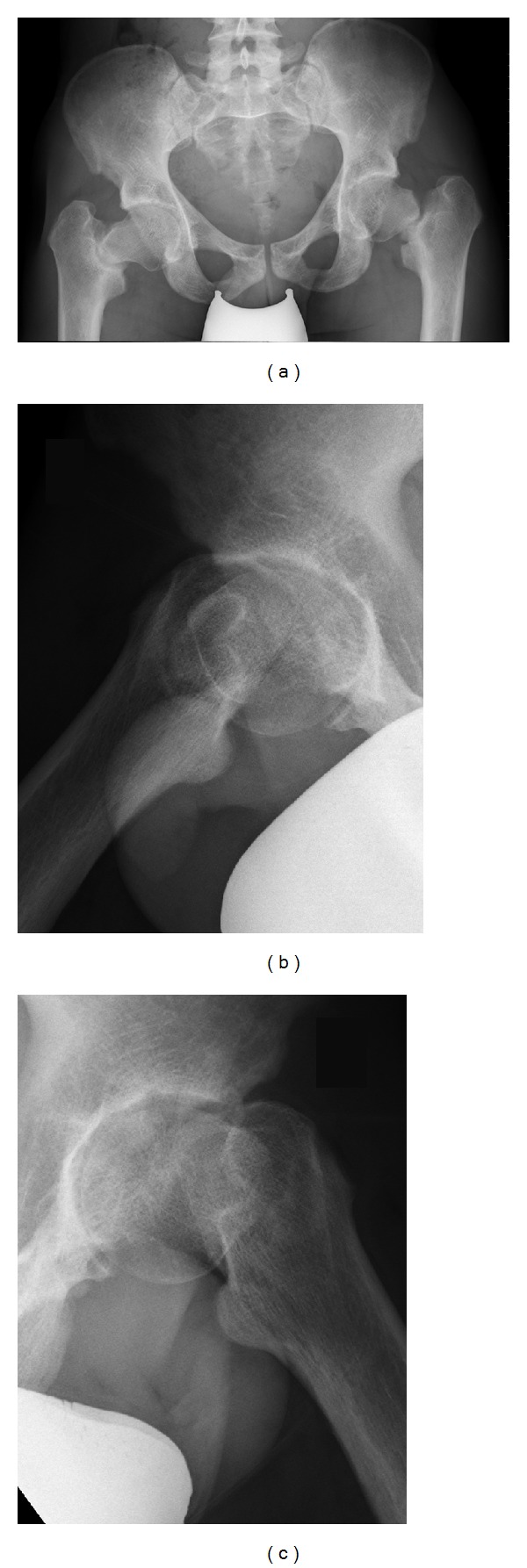
Standard pelvic radiograph showing bilateral pseudarthrosis of the femoral neck (a), axial radiograph of the right hip (b), and axial radiograph of the left hip (c).

**Figure 3:**
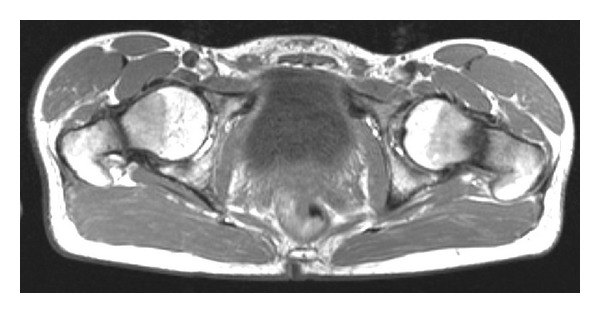
Axial T2-weighted MRI of the pelvis showing bilateral, atrophic nonunions of the femoral neck fractures.

Clinical examination revealed a patient with athletic build, weighting 53 kg and measuring 155 cm. Hip range of motion was (right side/left side) flexion/extension 120/0/0 and 130/0/0, endo-/exorotation 20/0/5 and 20/0/30, and ab-/adduction 10/0/30 and 15/0/30. Trendelenburg's sign was positive on both sides. The patient complained of pain by abduction and exorotation. The Harris and Oxford hip scores were 86 and 43, respectively. Haematological and biochemical parameters were within normal range except for a low phosphate of 1.7 mg/dL (norm: 2.3–4.7) and low potassium of 3.1 mmol/L (norm: 3.5–5.1). Radiographs of hips and pelvis (Figure 2) revealed fractures in both femoral necks. An MRI of the pelvis showed an atrophic nonunion of both fractures (Figure 3).

Treatment options were discussed with the patient and a conservative treatment was decided on. The patient was advised to participate in sports that did not involve shock loading of the hip joint, for example, swimming, cycling, and aquajogging. The patient was informed that a prosthetic replacement of the hip could become necessary by further increase of symptoms or necrosis of the femoral head.

## 3. Discussion

To our best knowledge, this patient represents the first case of bilateral femoral neck pseudarthrosis in HHR. HHR is a rare disease. However, some reports on stress fractures in these patients exist [8, 9]. In most of these reports, a stress fracture is described as subtrochanteric; probably due to the strong coxa vara deformity, a stress fracture of both femoral necks occurred, presenting as knee pain initially. An MRI of the hip at that stage would probably have demonstrated the lesion [10]. The natural history of stress fractures is the evolution towards a complete fracture. As the patient was not taking his prescribed medication regularly, we may speculate that this enhanced the formation of the pseudarthrosis.

Numerous surgical techniques have been described to treat pseudarthrosis of femoral neck fractures. Nevertheless, reports of successful management of femoral neck fatigue fracture nonunions are rare. The use of bone morphogenetic proteins [11], muscle pedicle grafts (Meyer's procedure) [12], valgus subtrochanteric osteotomies [13], and autologous bone graft harvesting from the iliac crest with cannulated screw fixation [14] have been described, respectively, in different case presentations all with good to excellent results. Arthroplasty is generally reserved for revision in young individuals. Displaced stress fracture fixation is known to have a high complication rate with revision required in up to 47% of patients, particularly if there is a delay in the diagnosis and treatment or if a pseudarthrosis has already formed [15]. In the case described here, a conservative treatment was chosen, knowing that revision and complication rates are very high even in patients with normal bone healing and because of the limited impairment in daily life activities and the mild pain. Also the history of multiple revisions, long immobility, and the pain the patient experienced after the osteotomies of both tibias ten years ago made the patient prefer a conservative treatment. When symptoms get worse in the future, prosthetic replacement of the joint seems the best possible treatment, as the risk of failure of osteosynthesis seems to be extremely high in this patient.
